# Cost Trend Analysis of Initial Cancer Treatment in Taiwan

**DOI:** 10.1371/journal.pone.0108432

**Published:** 2014-10-03

**Authors:** Tsai-Yun Li, Jan-Sing Hsieh, King-Teh Lee, Ming-Feng Hou, Chia-Ling Wu, Hao-Yun Kao, Hon-Yi Shi

**Affiliations:** 1 Department of General Affairs, Kaohsiung Medical University Hospital, Kaohsiung, Taiwan; 2 Department of Healthcare Administration and Medical Informatics, Kaohsiung Medical University, Kaohsiung, Taiwan; 3 Division of Gastrointestinal and General Surgery, Department of Surgery, Kaohsiung Medical University Hospital, Kaohsiung, Taiwan; 4 Division of Hepatobiliary Surgery, Department of Surgery, Kaohsiung Medical University Hospital, Kaohsiung, Taiwan; 5 Kaohsiung Municipal Ta-Tung Hospital, Kaohsiung, Taiwan; 6 National Sun Yat-Sen University-Kaohsiung Medical University Joint Research Center, Kaohsiung, Taiwan; 7 Institute of Clinical Medicine, Kaohsiung Medical University, Kaohsiung, Taiwan; 8 Department of Neurosurgery, Chimei Medical Center, Yongkang, Taiwan; University Hospital Heidelberg, Germany

## Abstract

**Background:**

Despite the high cost of initial cancer care, that is, care in the first year after diagnosis, limited information is available for specific categories of cancer-related costs, especially costs for specific services. This study purposed to identify causes of change in cancer treatment costs over time and to perform trend analyses of the percentage of cancer patients who had received a specific treatment type and the mean cost of care for patients who had received that treatment.

**Methodology/Principal Findings:**

The analysis of trends in initial treatment costs focused on cancer-related surgery, chemotherapy, radiation therapy, and treatments other than active treatments. For each cancer-specific trend, slopes were calculated for regression models with 95% confidence intervals. Analyses of patients diagnosed in 2007 showed that the National Health Insurance (NHI) system paid, on average, $10,780 for initial care of a gastric cancer patient and $10,681 for initial care of a lung cancer patient, which were inflation-adjusted increases of $6,234 and $5,522, respectively, over the 1996 care costs. During the same interval, the mean NHI payment for initial care for the five specific cancers increased significantly (p<0.05). Hospitalization costs comprised the largest portion of payments for all cancers. During 1996–2007, the use of chemotherapy and radiation therapy significantly increased in all cancer types (p<0.05). In 2007, NHI payments for initial care for these five cancers exceeded $12 billion, and gastric and lung cancers accounted for the largest share.

**Conclusions/Significance:**

In addition to the growing number of NHI beneficiaries with cancer, treatment costs and the percentage of patients who undergo treatment are growing. Therefore, the NHI must accurately predict the economic burden of new chemotherapy agents and radiation therapies and may need to develop programs for stratifying patients according to their potential benefit from these expensive treatments.

## Introduction

The growing incidence of cancer in aging populations and the use of new diagnostic technologies and targeted treatments are expected to result in increased cancer care costs. In the United States, the cancer incidence rate in patients aged 65 and older declined by 10% from 1992 to 2002. During the same period, however, the number of adults in this age group increased. Thus, the absolute number of people treated for cancer is projected to increase faster than the increase in the overall population [1]. According to the Taiwan Ministry of Health and Welfare, cancer has been one of the ten most common causes of death since 1982. In 2009, the five most common cancer types in Taiwan were lung cancer (19.9% of all cancer deaths), liver cancer (19.4%), colorectal cancer (11.4%), gastric cancer (5.7%), and female breast cancer (4.0%). Since 2010, oral cancer replaced gastric cancer as the fourth most common cancer type [1], [2]. These high incidence and mortality rates result in major medical expenditures and large socioeconomic impacts on patients, their families, and the society as a whole.

Analyses of cancer-related costs are usually performed in three phases to reflect clinical and cost-related dynamics: initial phase (the time following diagnosis, usually 1 year after diagnosis), continuing phase (all time occurring between initial and final phase) and final phase (the time before death, usually 1 year before death) [3]. Previous studies of cancer care costs have shown that a sizeable portion of cancer care costs are incurred in the initial phase [4], [5]. Taiwan studies of cancer costs have been limited to specific expenditures or to specific disease phases. Some Taiwan studies have also analyzed the cost effectiveness of specific cancer screening programs designed for early detection of cancers [6], [7]. Such studies analyze macroeconomic data related to specific procedures.

Therefore, the purpose of this study was to estimate cost trends in initial cancer care during 1996–2007. The analysis focus on patients diagnosed with breast, colorectal, liver, lung, or gastric cancer since these cancers comprise approximately 60% of all cancers in the nationwide population [2]. Despite the high cost of initial cancer care, data for specific categories of cancer-related expenditures, especially costs of specific services, are limited. This study analyzed cost trends in specific healthcare services (surgery, chemotherapy, radiation therapy, and other treatments) as well as overall cost trends. We hypothesized that the increases in initial care costs reflect both increased rates of treatment for cancer patients and increased costs of specific therapies.

## Materials and Methods

### Data Source

The National Health Insurance (NHI) claims database includes data on outpatient visits, hospital admissions, prescriptions, disease, and vital statistics for 99% of the national population of 23 million. This study performed a longitudinal analysis of the medical history of each beneficiary by linking several claims datasets and the National Death Registry. Since its establishment in March 1, 1995, the single-payer NHI program has provided universal coverage and equal access to health-care services in Taiwan. The National Health Research Institute (NHRI) maintains the NHI Research Database (NHIRD) based on data provided by the Bureau of National Health Insurance (BNHI). This large computerized database, which is open to researchers in Taiwan, contains registration files and original claims data for reimbursement under NHI programs [8]. Therefore, it offers researchers a representative nationwide database for the Taiwan health-care system. The sub-dataset used in this study comprised 1 million patients randomly selected from the 23 million insurants who registered during 1996-2000. For each insurant, this database contained all medical records for 1996–2010, including diagnoses coded according to the International Classification of Diseases, 9th Revision, Clinical Modification (ICD-9-CM).

### Sample Selection

The NHRI database was searched to identify all patients diagnosed with lung cancer (ICD-9 code 162.0–162.9), liver cancer (ICD-9 code 155.0–155.2), colorectal cancer (ICD-9 code 153.0–154.8), gastric cancer (ICD-9 code 151.0–151.9), or female breast cancer (ICD-9 code 174.0–174.9) during the period from January, 1996 to December, 2007. Patients were excluded if the month of diagnosis was unknown or if they had been identified by the BNHI registry through a death certificate or an autopsy. To capture all services provided, the analysis was limited to patients enrolled in the NHI cost-of-care program in the first year after diagnosis. Additionally, to avoid capturing treatment costs for more than one cancer type, patients were only included if the NHRI data showed no prior or subsequent cancer treatment.

### Defining Cost of Care

In this study, outpatient and inpatient data for cancer treatment claims were merged with the cancer registry dataset in order to profile the long-term costs of treating individual cancer patients. Care costs were estimated by a phase-of-care approach [3]. The initial phase was defined as the first 12 months after a cancer diagnosis. Additionally, other Taiwan studies of care costs have similarly used reimbursement, rather than charges, as a proxy for medical care costs because the NHI charge for a service is not necessarily related to the actual cost of providing the service [9]–[11]. In contrast, NHI costs, which are typically defined as actual payments derived from reimbursement formulas, are used to reflect the average resource utilization for a health service [11], [12].

The Taiwan BNHI claims data were analyzed to determine the following hospital treatment costs: operating room, radiology, physical therapy, hospital room, anesthetist, pharmacy, laboratory, special materials, surgeon, and others. Hospital treatment cost was also adjusted for specific hospital levels according to differences in BNHI reimbursements. To reflect changes in real dollar value, cost data were also adjusted by the consumer price index for each year during 1996–2007. Finally, hospital treatment costs were converted from Taiwan dollars to US dollars at a ratio of 30:1, which was the average exchange rate during 1996–2007.

To capture all care costs associated with the initial diagnosis and treatment of cancer, this study defined initial care as care provided from immediately before diagnosis through 365 days after diagnosis. The date of diagnosis was defined as the first day of the month of diagnosis unless the NHI data revealed a cancer surgery in the month before the date of diagnosis indicated in the NHI database. In such cases, the date of surgery entered in the NHI database was used as the date of diagnosis.

The analysis of trends in initial treatment costs focused on cancer-related surgery, chemotherapy, radiation therapy, and treatments other than active treatments. The categories were defined as follows. Cancer-related surgery costs were assessed beginning from the date of surgery, as reported in the NHI claim, until the end of the cancer-related surgery period. The cancer-related surgery period was determined according to the intensity of the surgical procedure, and all NHI claims during this period were included in the estimate of cancer-related surgery costs. In the case of multiple procedures (e.g., modified radical mastectomy followed by breast conservation surgery), a hierarchy of surgical procedures was used to identify the most invasive surgical procedure that was cancer-related. The period associated with each surgery and the hierarchy of procedures were based on input from two authors who were experienced surgeons (K. T. Lee and M. F. Hou). Radiation therapy included both neo adjuvant therapy and adjuvant treatment. Costs were identified by reviewing the physician file. Claims for radiation therapy were also included if they had been reported in the hospital outpatient and hospital inpatient files. If an outpatient claim included radiation therapy along with other services, the radiation therapy was excluded since the claims only indicated the total payment. Thus, in these cases, radiation therapy costs were indistinguishable from the costs of other services. Chemotherapy costs were defined as all NHI payments, excluding radiation therapy claims, incurred from the date of the first chemotherapy claim to the date of the last chemotherapy claim. The rationale for including all NHI payments other than those for radiation is that most of the care received by a patient undergoing chemotherapy is related to either administration of chemotherapy or to monitoring and treatment of its effects. For persons who had received cancer-related surgery, chemotherapy costs included any neo adjuvant or adjuvant therapy received after the end of the cancer-related surgery period. Other treatment costs were defined as any NHI payments for inpatient stays other than those in the time windows for cancer-related surgery or chemotherapy.

### Statistical Analysis

The unit of analysis in this study was the individual cancer patient undergoing treatment. Trends were calculated based on estimates for successive calendar years. Temporal trends in initial treatment costs were assessed by the Cochrane-Armitage trend test. For patients whose care spanned more than one calendar year, all costs were assigned to the year of diagnosis. To identify causes of changes in costs over time, cost trends were assessed according to two parameters: the percentage of patients who had received a specific treatment (i.e., surgery, chemotherapy, radio therapy, or other treatment) and the mean cost of care associated with that treatment. Linear regression models were used to determine whether the cost trends and the proportion of treated patients significantly differed. In these models, the independent variable was the calendar year, and the dependent variables were the percentage of patients treated and the treatment costs. The slope of regression models with 95% confidence intervals (95% C.I.) was calculated for each cancer-specific trend.

For breast cancer, only the gender-specific population was analyzed. To determine the costs of specific service categories, the total number of beneficiaries was estimated for each service. For each cancer site, this estimate was derived by calculating the proportion of patients who had received the service and then multiplying the proportion by the total number of beneficiaries diagnosed with breast, colorectal, liver, lung, or gastric in 2007. The total NHI payment for the year 2007 patients was then calculated by multiplying the estimated 2007 mean payment for the service by the estimated total number of NHI fee-for-service beneficiaries who had received the service.

Statistical analyses were performed with SPSS, version 18.0 (SPSS Inc., Chicago, IL, USA). All tests were two-sided, and p values less than 0.05 were considered statistically significant.

## Results

**Figure 1:**
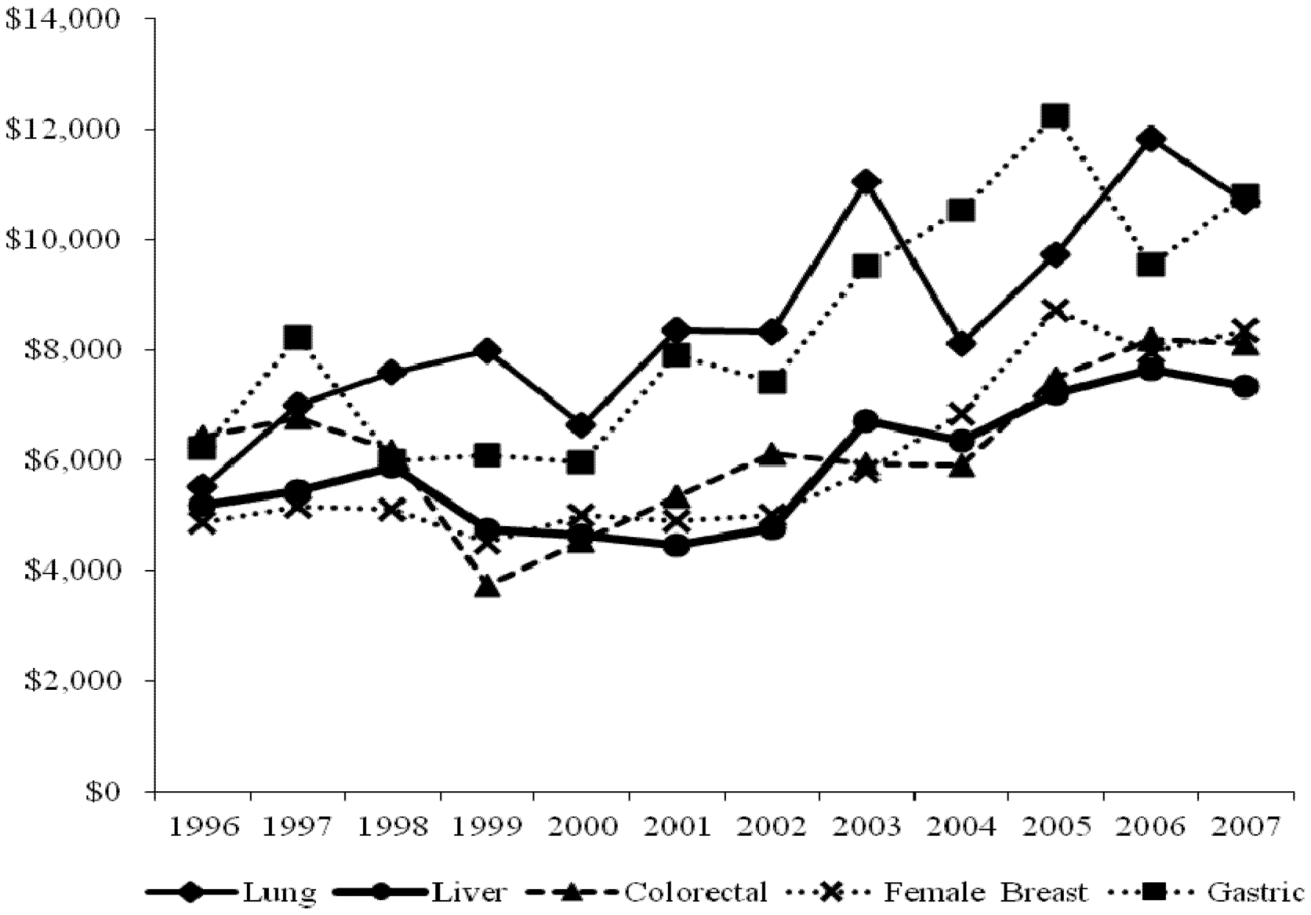
Trends in the cost of initial cancer treatment from 1996 to 2007.

The cohort in this study included 141,772 fee-for-service NHI beneficiaries diagnosed with lung cancer, liver cancer, colorectal cancer, gastric cancer, or female breast cancer during 1996-2007 (Table 1), which was the period during which the initial care costs for these five specific cancers significantly increased (p<0.05). In each year, care for gastric and lung cancer care had the largest mean NHI payments and the largest payment increase in terms of absolute dollars (Figure 1). In 2007, the average costs for initial care for gastric cancer and lung cancer were $10,780 and $10,681, respectively. These costs were $4,546 and $5,159 higher than the average costs for gastric cancer and lung cancer, respectively, in 1996. The mean payment for breast cancer rose from $4,876 in 1996 to $8,347 in 2007. During the same interval, the mean payment for colorectal cancer increased from $6,433 to $8,113, and the mean payment for liver cancer increased from $5,179 to $7,347.

**Table 1:** Comparison of five different cancers in terms of mean costs in initial phase of treatment (including surgery, chemotherapy, radiation therapy, and other treatment) from 1996 to 2007.^a^

$(USD)		1996	1997	1998	1999	2000	2001	2002	2003	2004	2005	2006	2007
Lung cancer	Total cost	5,522	7,005	7,595	8,000	6,634	8,356	8,338	11,056	8,114	9,735	11,819	10,681
	Surgery	940	936	944	888	920	770	898	1,067	1,166	1,070	1,338	1,495
	Chemotherapy	673	905	3,346	2,833	2,103	3,503	4,044	3,949	3,026	5,102	4,973	4,625
	Radiation therapy	1,132	2,318	2,199	2,312	2,283	3,497	2,761	4,388	3,648	3,740	2,756	4,070
	Other treatment	4,908	2,927	3,439	4,745	3,502	4,000	4,682	8,682	4,348	6,034	8,350	5,600
Liver cancer	Total cost	5,179	5,458	5,894	4,756	4,650	4,457	4,767	6,718	6,349	7,212	7,659	7,347
	Surgery	514	829	1,075	960	1,080	1,176	1,042	1,285	1,239	1,266	1,012	1,068
	Chemotherapy	406	282	326	499	474	236	264	621	1,427	484	979	820
	Radiation therapy	2,115	2,045	2,185	1,516	1,900	1,724	3,441	4,115	2,781	5,512	6,664	4,607
	Other treatment	4,810	3,810	5,058	3,815	3,647	2,955	3,432	5,047	4,300	5,283	5,478	4,645
Colorectal cancer	Total cost	6,433	6,778	6,182	3,747	4,528	5,333	6,116	5,949	5,917	7,486	8,201	8,113
	Surgery	941	794	737	788	835	880	934	921	1,058	1,281	1,253	1,330
	Chemotherapy	969	397	702	1,129	1,396	1,375	2,935	2,121	2,320	3,908	3,726	3,437
	Radiation therapy	1,353	2,112	2,810	3,797	3,076	3,587	4,589	3,778	6,787	5,459	7,998	4,377
	Other treatment	5,194	4,271	4,132	2,296	2,919	2,935	2,420	2,458	2,770	3,228	3,990	3,641
Breast cancer	Total cost	4,876	5,162	5,104	4,512	5,015	4,902	4,996	5,805	6,856	8,707	7,959	8,347
	Surgery	417	411	423	426	390	417	428	362	460	437	453	478
	Chemotherapy	3,152	2,325	2,133	1,551	1,694	1,504	2,416	2,021	4,264	4,225	2,913	3,650
	Radiation therapy	2,278	2,328	2,656	2,524	2,962	3,828	3,297	2,979	4,073	5,095	5,246	6,310
	Other treatment	3,411	724	1,247	2,229	1,973	1,396	1,062	1,109	1,462	1,801	896	1,813
Gastric cancer	Total cost	6,234	8,231	5,995	6,087	5,977	7,918	7,426	9,529	10,533	12,254	9,541	10,780
	Surgery	930	944	916	983	926	1,029	994	1,202	1,125	1,078	1,063	1,180
	Chemotherapy	337	1,068	1,267	1,036	1,448	2,129	1,036	2,062	823	1,484	3,481	2,717
	Radiation therapy	1,380	1,693	1,066	1,775	2,681	2,540	1,774	1,814	3,117	7,068	6,069	7,741
	Other treatment	5,861	3,120	3,682	3,856	2,125	4,867	4,911	4,085	5,506	9,776	4,227	7,624

a Other treatment: Any non-active treatment other than surgery, chemotherapy or radiation therapy.

**Figure 2:**
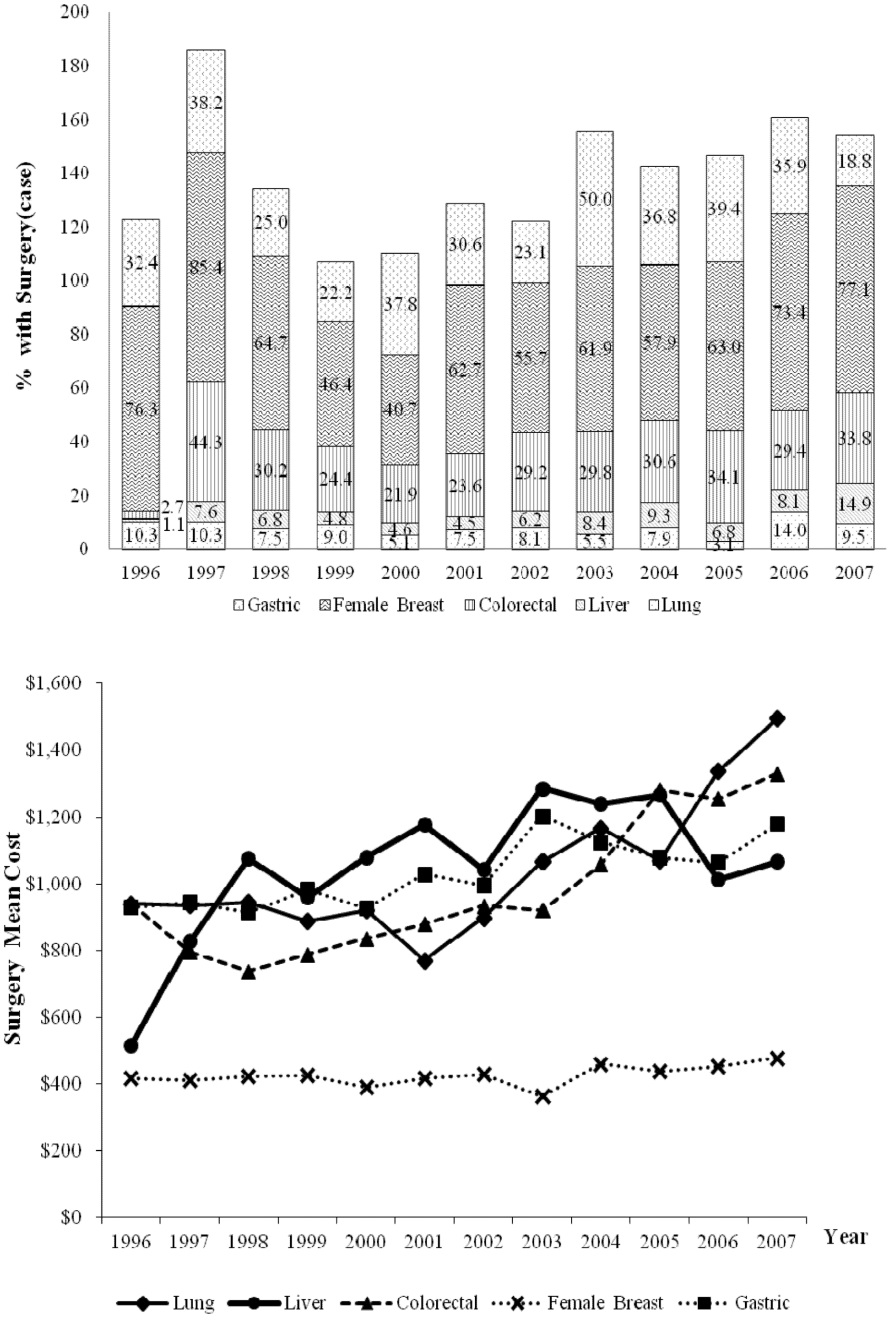
Trends in the percent of NHI beneficiaries undergoing cancer-related surgery and mean costs of initial cancer treatment from 1996 to 2007.

The percentage of patients undergoing cancer-related surgery varied by cancer site (Figure 2). In each year analyzed, at least 60% of breast cancer patients underwent surgery. In colorectal and liver cancer patients, 2.7% and 1.1%, respectively, were surgically treated in 1996 whereas 33.8% and 14.9%, respectively were surgically treated in 2007. Therefore, the percentage of patients undergoing cancer-related surgery significantly increased in these two cancer types during the study period (yearly rate of increase = 2.8%, 95% CI = 1.2% to 4.4% in colorectal cancer and 1.2%, 95% CI = 0.4% to 2.0% in liver cancer). During the same period, lung cancer had the largest increase in mean cost of surgery (increase of $555 or 59.0%). In 2007, the mean cost of surgery was the highest for lung cancer ($1,495) followed by colorectal ($1,330), gastric ($1,180), liver ($1,068), and breast ($478) cancers.

**Figure 3:**
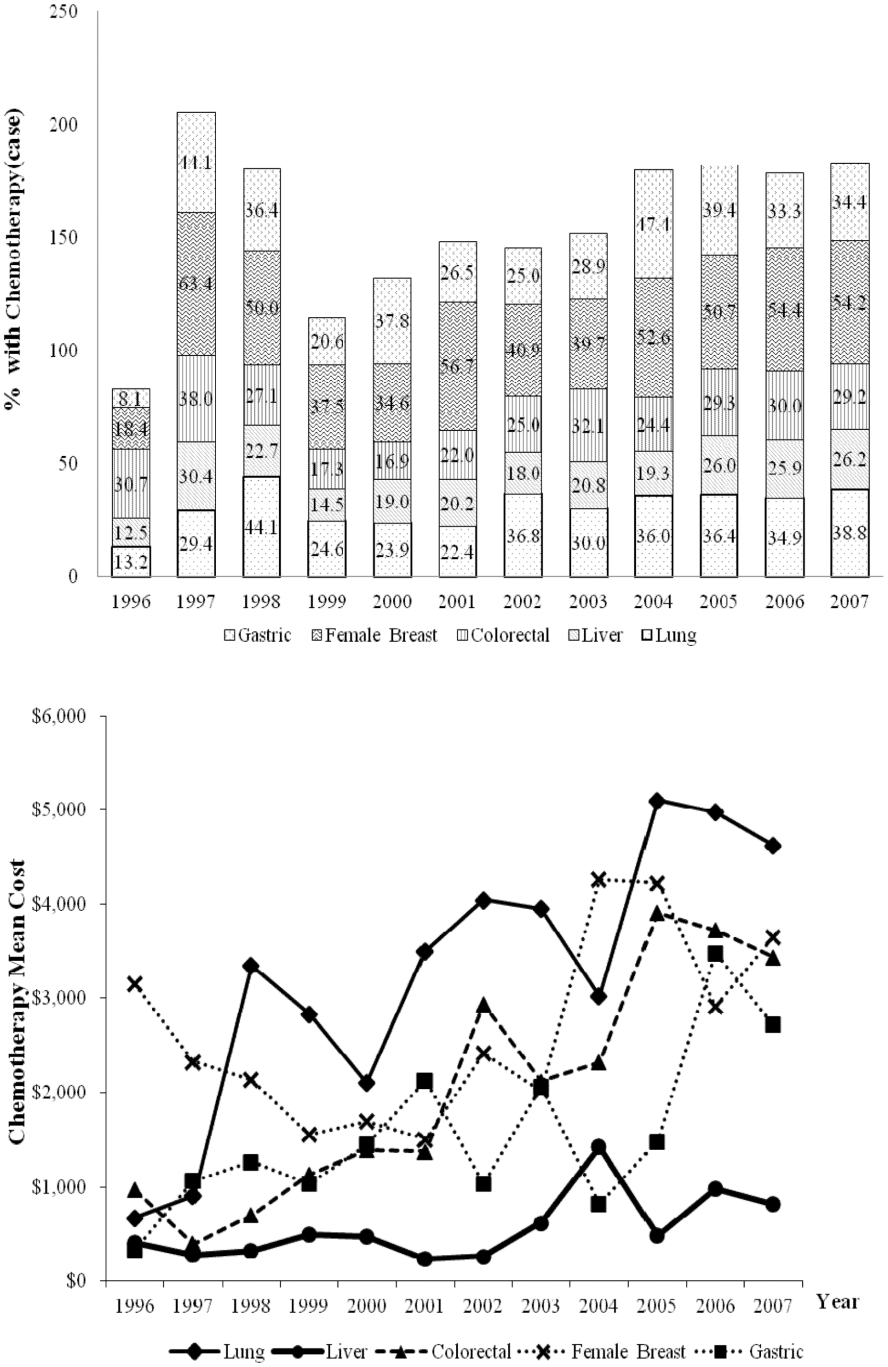
Trends in the percent of NHI beneficiaries receiving chemotherapy and mean costs of initial cancer treatment from 1996 to 2007.

In all years analyzed, chemotherapy was performed in at least 25%, 40%, and 20% of patients with gastric, breast, and colorectal cancers, respectively (Figure 3). In 1996, chemotherapy was performed in 8.1%, 18.4%, 12.5%, and 13.2% of patients with stomach, breast, liver, and lung cancer, respectively. By 2007, the respective percentages had significantly increased to 34.4%, 54.2%, 26.2%, and 38.8%. The yearly rates of increase were 2.4% for gastric cancer (95% CI = 1.0% to 3.9%), 3.3% for breast cancer (95% CI = 1.7% to 5.5%), 1.2% for liver cancer (95% CI = 0.6% to 1.8%) and 2.3% for lung cancer (95% CI = 1.3% to 3.4%). During the same period, the mean cost of chemotherapy for lung cancer patients increased by $3,952 (587.2%). This increase exceeded that for all other cancer types. In 2007, the mean cost of chemotherapy was highest for lung cancer ($4,625) followed by breast ($3,650), colorectal ($3,437), gastric ($2,717), and liver ($820) cancers.

**Figure 4:**
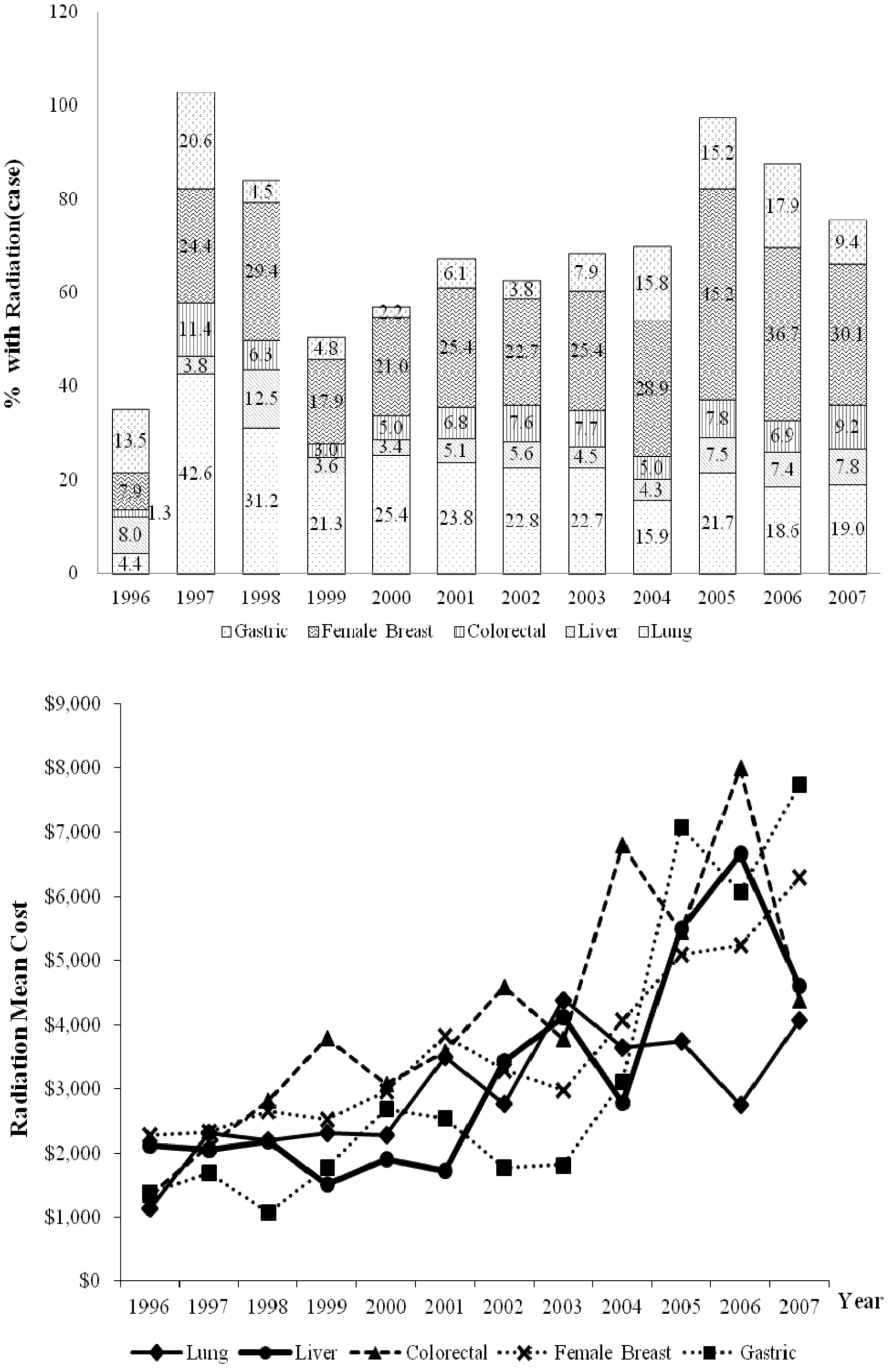
Trends in the percent of NHI beneficiaries receiving radiation therapy and mean costs of initial cancer treatment from 1996 to 2007.

In all years analyzed, radiation therapy was received by at least 25% and 20% of patients with breast cancer and lung cancer, respectively (Figure 4). In 1996, radiation therapy was received by 7.9%, 1.3%, and 4.4% of patients with breast, colon and lung cancers, respectively. In 2007, the respective figures significantly increased to 30.1%, 9.2%, and 19.0%. The yearly rates of increase were 2.4% for breast cancer (95% CI = 1.2% to 3.6%), 0.7% for colorectal cancer (95% CI = 0.1% to 1.4%), and 1.3% for lung cancer (95% CI = 0.5% to 2.2%). During the same period, gastric cancer had the largest increase in the mean cost of radiation therapy ($7,741 or 460.9%). In 2007, the mean cost of radiation therapy was highest for gastric cancer ($7,741) followed by breast ($6,310), liver ($4,607), colorectal ($4,377), and lung ($4,070) cancers.

**Figure 5:**
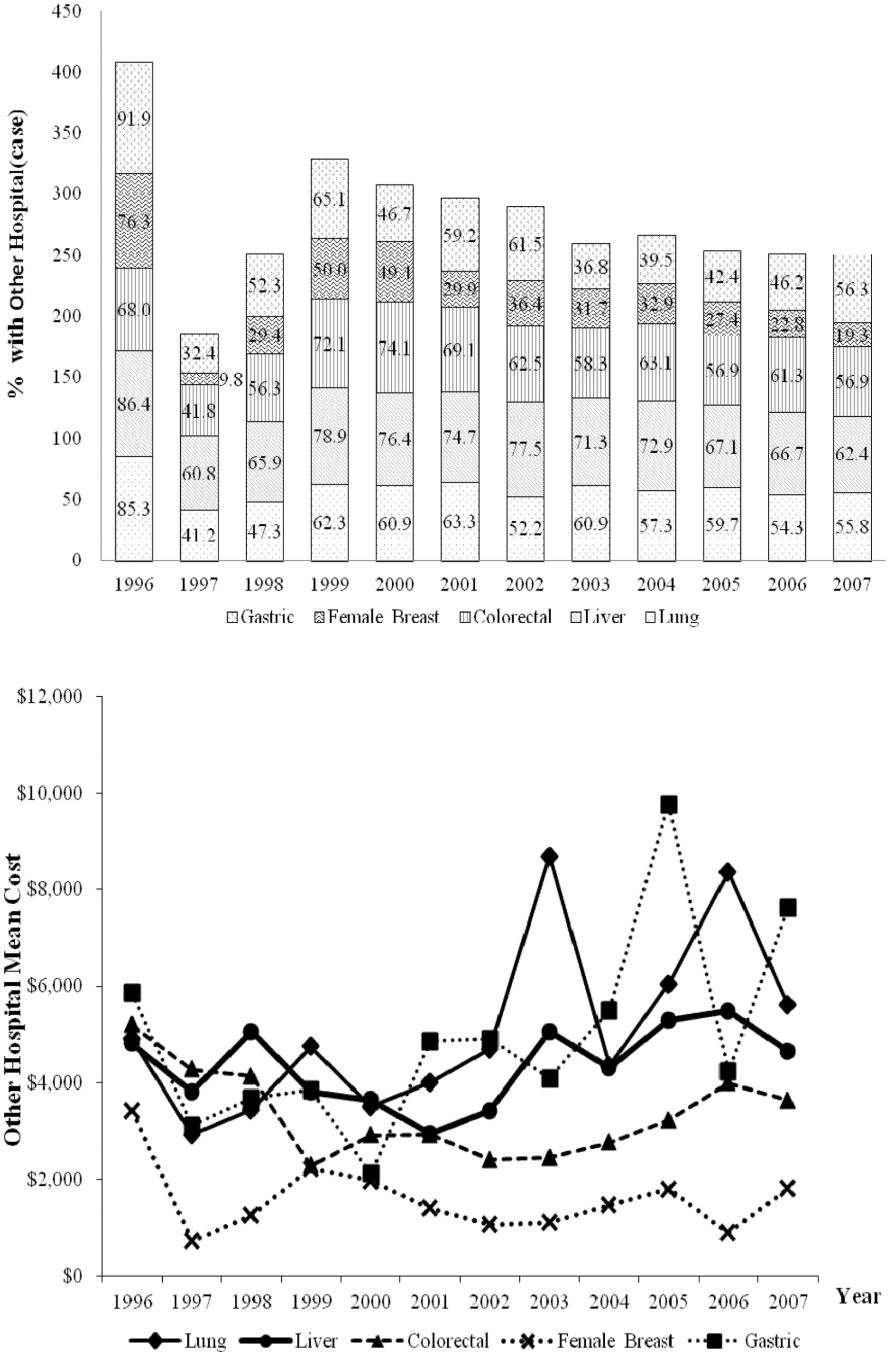
Trends in the percent of NHI beneficiaries with other treatment (no active treatment) and mean costs of initial cancer treatment from 1996 to 2007.

In all years analyzed, treatments other than active treatments were received by more than 40%, 55%, 60%, and 50% of patients with gastric, colorectal, liver, and lung cancers, respectively (Figure 5). In 1996, treatments other than active treatments were received by 91.9%, 76.3%, 86.4%, and 85.3% of patients with gastric, breast, liver, and lung cancers, respectively. In 2007, the respective figures were 56.3%, 19.3%, 62.4%, and 55.8%, which were statistically significant decreases. The yearly rates of decrease were 3.2% for gastric cancer (95% CI = 1.4% to 5.0%), 5.2% for breast cancer (95% CI = 3.1% to 7.2%), 2.2% for liver cancer (95% CI = 1.1% to 3.2%), and 2.7% for lung cancer (95% CI = 1.3% to 4.1%). During the same period, gastric cancer patients showed the largest increase in the mean cost of treatment other than active treatment ($1,763; 30.1%), and colorectal cancer patients showed the largest decrease ($1,553 or 29.9%). In 2007, the mean cost of treatment other than active treatment was highest for gastric cancer ($7,624) followed by lung ($5,600), liver ($4,645), colorectal ($3,641), and breast ($1,813) cancers.

## Discussion

To the best of our knowledge, this population-based study is the first to assess nationwide trends in the cost of initial cancer care in Taiwan. As health-care costs continue to increase, understanding cost trends in health care and identifying contributing factors in increased treatment costs will be important for planning for future health-care costs and for prioritizing and allocating medical resources. This study revealed significant increases in the mean total cost incurred by the NHI for treating patients with lung, liver, colorectal, breast, or gastric cancers in the initial period after diagnosis. Since these trend estimates provide working assumptions about health service costs and dissemination, health investigators can use these baseline data to model complex cost structures of specific emergency technologies and medical practices. This Taiwan study revealed increases in the mean total cost of initial treatment for five specific cancers during 1996–2007. This result, which is consistent with previous studies, may be due to an aging trend in the overall population and to an increased survival rate for these specific cancers as a result of improvements in health-care treatment models, medical utilization, high-technology equipment, and payment systems [13]–[15].

In a review of cancer studies performed in the United States, Warren et al. [15] reported statistically significant increases in initial cancer care costs. The main causes of the increase were increases in the numbers of patients receiving surgery and adjuvant therapy and increases in the costs of these treatments. Although expected these trends to continue in the near future, they argued that the overall economic impact of this trend can be mitigated by targeting cost reduction measures at the most costly therapies. Studies of cancer care costs over multiple phases of care generally agree that cancer-related costs and total costs exhibit a u-shaped curve, i.e., costs are generally highest in the initial phase and then decrease in the continuing phase [16]–[18]. These findings were consistent across all cancer types evaluated here. Additionally, other studies that have evaluated care costs for multiple cancer types using the same data source and methods generally show that, for each phase of care, total costs and cancer-related costs are generally higher for lung cancer than for breast and prostate cancers [16]–[18].

All five cancer types analyzed in the present study revealed significant increases in the proportions of patients treated with adjuvant chemotherapy and radiation therapy. These increasing rates may result from the accumulation of NHI data for the response of type-specific cancer patients to these adjuvant treatments, which the NHI uses to estimate the survival benefit of the treatments. Another contributing factor is the publication of studies throughout the study period confirming the benefit of chemotherapy and radiation therapy for lung cancer [19], [20]. Additionally, patients with lung cancer reportedly have a longer mean length of stay in the initial phase of care compared to patients with breast, colorectal, or other gastrointestinal cancers [21]. Given the dynamic nature of the health-care delivery system in recent decades and the rapid innovations in cancer treatment, consistent time trends must also be identified for specific categories of cancer care, e.g., surgery, other in-patient care, chemotherapy, radiation therapy, supportive care, and hospice care [19]–[21].

In the NHI scheme in Taiwan, reimbursement claims for cancer diagnosis and treatment were the second highest of all major illnesses and injuries [1]. A method of accurately estimating cancer care costs in Taiwan is urgently needed because all medical costs related to cancer diagnosis and management are fully reimbursed by the NHI. Mariotto et al. [14] linked the Surveillance, Epidemiology, and End Results database to Medicare records to estimate costs in the initial, continuing, and final phases of care for 13 cancers in men and 16 in women. They projected a 27% increase in national costs (to $157.77 billion) by 2020 in the base scenario (assuming constant incidence, survival, and cost). However, they also concluded that, despite the inevitable increases in cancer care costs as the population ages, effective management of the costs of new treatments and diagnostic technologies can ensure access to quality care for all patients. In Yabroff et al. [22], a descriptive review of the literature on cancer care costs in the US revealed wide variation in study settings, populations, services types included, cost measurements, and study methods. Nevertheless, all studies consistently showed increasing costs of cancer care.

The present study revealed a dramatic increase in the average payment for adjuvant treatments and an increase in the percentage of cancer patients treated with chemotherapy. In the mid-to-late 1990s, the use of new and expensive agents (e.g., paclitaxel, docetaxel, and gemcitabine), administered alone or in combination with existing agents showed superior survival benefits compared with prior approaches [23]. However, the costs of these agents can be substantial. Given that many of the new and expensive targeted therapies are combined with existing therapies, they may add to rather than substitute for adjuvant therapy costs. Therefore, these new agents may be a large contributor to the rapidly escalating costs of cancer care. Another contributing factor in the rising costs of chemotherapy is the increased use of erythropoiesis-stimulating agents and granulocyte colony-stimulating factor (G-CSF) [24]. However, the costs of erythropoiesis-stimulating agents and G-CSF may be justifiable if they enable the patient to maintain the treatment schedule.

A limitation of studies use diagnostic or procedure codes from reimbursement claims to identify patients with incident disease is that they identify prevalent cases, over-identify cases from rule-out diagnostic procedures, and under-identify patients who have not received specific procedures or treatments due to insufficiently detailed coding [25], [26]. The diagnostic codes might also reflect metastatic rather than primary tumor sites. The phase-of-care approach classifies cancer patients according to the time of diagnosis and survival time. However, this classification was often problematic in the studies reviewed here. Another concern is the limitations of the method used to analyze and report cost data, such as methods for addressing censored or missing data and skewed distributions of cost data. Although other studies have proposed standards for conducting and reporting cost-effectiveness analyses, no studies have proposed standards for conducting and reporting cost analyses [27], [28]. This issue merits further attention in future studies.

In addition to exclusion of the most recently introduced chemotherapy agents, other limitations of this study are noted. For patients identified as undergoing cancer-directed surgery, the NHI procedures reported on the claims forms could have been miscoded. However, previous analyses of NHI data for specific surgical procedures agree that NHI data tend to be highly accurate and reliable. Moreover, this study did not include cancer stage, which is an important consideration in the choice of initial treatment, because cancer stage was not included in the NHI database. The cost estimates in this study are incomplete in several ways. Some patients did not receive cancer-related treatment according to the NHI data. The percentage of these patients varied by cancer site, which may explain why patients who presented with advanced disease did not undergo curative care. Many patients who did not receive cancer-directed treatment were hospitalized throughout the year. Therefore, their treatment costs would have been captured in the “other treatment” category.

Apart from the growing number of NHI beneficiaries with cancers, the percentage of patients undergoing treatment is increasing, and treatment costs are rising. In 2007, the total NHI expenditures for initial care exceeded $12 billion for the five specific cancers analyzed in this study. These data do not reflect the current (2014) or future cancer-care-related costs to the NHI program. Since expensive adjuvant therapies are expected to strain the financial resources of the NHI program, the Ministry of Health and Welfare must anticipate the economic burden of new chemotherapy agents and radiation therapies and may need to develop programs for identifying the patients who would receive the greatest benefit from these expensive treatments.
